# HSP-70-Mediated Hyperbaric Oxygen Reduces Brain and Pulmonary Edema and Cognitive Deficits in Rats in a Simulated High-Altitude Exposure

**DOI:** 10.1155/2018/4608150

**Published:** 2018-11-01

**Authors:** Hsing-Hsien Wu, Ko-Chi Niu, Cheng-Hsien Lin, Hung-Jung Lin, Ching-Ping Chang, Chia-Ti Wang

**Affiliations:** ^1^Division of Thoracic Surgery, Department of Surgery, Tainan Municipal Hospital (Managed by Show Chwan Medical Care Corporation), Tainan, Taiwan; ^2^Department of Nursing, Chung Hwa University of Medical Technology, Tainan, Taiwan; ^3^Department of Hyperbaric Oxygen, Chi Mei Medical Center, Tainan, Taiwan; ^4^Department of Medicine, Mackay Medical College, New Taipei, Taiwan; ^5^Department of Emergency Medicine, Chi Mei Medical Center, Tainan, Taiwan; ^6^Department of Biotechnology, Southern Taiwan University of Science and Technology, Tainan, Taiwan; ^7^Department of Medical Research, Chi Mei Medical Center, Tainan, Taiwan

## Abstract

High-mountain sickness is characterized by brain and pulmonary edema and cognitive deficits. The definition can be fulfilled by a rat model of high-altitude exposure (HAE) used in the present study. This study aimed to investigate the protective effect of hyperbaric oxygen therapy (HBO_2_T) and to determine the underlying mechanisms. Rats were subjected to an HAE (9.7% O_2_ at 0.47 absolute atmosphere of 6,000 m for 3 days). Immediately after termination of HAE, rats were treated with HBO_2_T (100% O_2_ at 2.0 absolute atmosphere for 1 hour per day for 5 consecutive days) or non-HBO_2_T (21% O_2_ at 1.0 absolute atmosphere for 1 hour per day for 5 consecutive days). As compared to non-HAE+non-HBO_2_T controls, the HAE+non-HBO_2_T rats exhibited brain edema and resulted in cognitive deficits, reduced food and water consumption, body weight loss, increased cerebral inflammation and oxidative stress, and pulmonary edema. HBO_2_T increased expression of both hippocampus and lung heat shock protein (HSP-70) and also reversed the HAE-induced brain and pulmonary edema, cognitive deficits, reduced food and water consumption, body weight loss, and brain inflammation and oxidative stress. Decreasing the overexpression of HSP-70 in both hippocampus and lung tissues with HSP-70 antibodies significantly attenuated the beneficial effects exerted by HBO_2_T in HAE rats. Our data provide in vivo evidence that HBO_2_T works on a remodeling of brain/lung to exert a protective effect against simulated high-mountain sickness via enhancing HSP-70 expression in HAE rats.

## 1. Introduction

High-mountain sickness characterized by cerebral and pulmonary edema and cognitive dysfunction that occurs after high-altitude exposure (HAE) is commonly seen among climbers and tourists [1, 2]. Indeed, the high-mountain sickness syndromes displayed by patients can be reproduced by the rats exposed to HAE [3, 4].

Hyperbaric oxygen (HBO_2_) preconditioning has been promoted as a promising method for preventing the occurrence of cerebral and pulmonary edema in rats exposed to a simulated HAE [4, 5]. However, it is not known whether HBO_2_ adopted after a simulated HAE is still able to ameliorate the occurrence of cerebral and pulmonary edema and its underlying mechanisms.

It is well accepted that heat shocks protein- (HSP-) 70 induction by several physiological or pathological stress protects against subsequent damage by increasing the tolerance of affected cells [6, 7]. Full protection may not be observed if HSP-70 expression is at reduced levels or if the insult is severe [6, 7]. Although a recent study [8] has demonstrated that decreasing or increasing HSP-70 exacerbates or attenuates heat-induced cell death, respectively, in rat hypothalamic cells, a recent report indicates that HSP-70 is a novel biomarker reflecting the severity of disease conditions [9]. Therefore, further studies are needed to establish the role of HSP-70 activity in the pathogenesis of high-mountain sickness.

To date, no studies have explored the potential of hyperbaric oxygen therapy (HBO_2_T) to protect animals in the setting of high-mountain sickness characterized by cerebral and pulmonary edema with its complications. In this study, we attempt to investigate the hypothesis that HBOT can induce protective properties in an animal model of high-mountain sickness via stimulating HSP-70 activity.

## 2. Materials and Methods

### 2.1. Ethical Approval of the Study Protocol

All the experiments were approved by the ethical guidelines laid down by The Institutional Animal Care and Use Committee at Chi Mei Medical Center (IACUC approved number: 101122406-1). All protocols were designed to minimize pain and discomfort during both the injury procedure and recovery.

### 2.2. Animals and Experimental Groups

**Figure 1:**
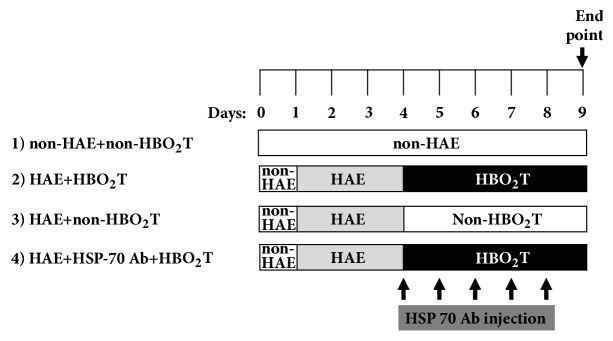
Experimental design: HAE: 9.8% O_2_ at 0.47 ATA; non-HAE: 21% O_2_ at 1.0 ATA; HBO_2_T: 100% O_2_ at 2.0 ATA 1 h per day; and non-HBO_2_T: 21% O_2_ at 1.0 ATA.

Adult male Wistar rats (280-300 g at the beginning of the study) were maintained at 22±1.0°C with 12:12 h light to dark cycle and given food and water* ad libitum*. One hundred and twenty rats were randomly assigned to one of the following 4 groups: (i) non-HAE+non-HBO_2_T group: the rat is exposed to normobaric air (non-HBO_2_T: 21% at 1.0 ATA) throughout the entire experiments; (ii) HAE+non-HBO_2_T group: immediately after HAE, the rat is treated with normobaric air; (iii) HAE+HBO_2_T group: immediately after high-altitude exposure (HAE: 9.8% O_2_ at 0.47 ATA for 3 days), the rat is treated with one regimen of HBO_2_T (100% O_2_ at 2.0 ATA 1 h per day for 5 consecutive days); and (iv) HAE+HSP-70Ab+HBO_2_T group: the rat is treated with one regimen of both HSP-70Ab HBO_2_T immediately after HAE and once daily for 5 consecutive days as summarized in Figure 1. Figure 1 illustrates the timeline of experimental procedures. On Day 9, after the last HBO_2_T, the rats were deeply anaesthetized with sodium pentobarbital (65 mg/kg, intraperitoneally; Sigma-Aldrich, St. Louis, MO, USA) and both the blood and brain samples were collected for verification.

### 2.3. A Simulated High-Mountain Sickness Model

There is no precise method for modelling high-mountain sickness in animals [10]. In the present study, we modified our previous study [9] by exposing rats to a simulated HAE (9.7% O2 at 0.47 ATA) of 6,000 m in a hypobaric chamber for 3 days to induce a simulated high-mountain sickness. In accordance with the study of Patir et al. [11], we evaluated tissue water content and vascular leakage, two critical indicators of cerebral and lung edema.

### 2.4. Hippocampal and Lung Expression Levels of HSP-70 and Inhibition of HSP-70 Activity

Anaesthetized rats were killed by decapitation at the end of the experiment, and their brains and lungs were immediately removed. The hippocampus was separated from the brain. After carefully rinsing in cooled phosphate-buffered saline, the tissues were homogenized on ice in a RIPA (Radio Immuno Precipitation Assay) lysis buffer (Bio-Rad Laboratories, Inc., Hercules, CA, USA). The hippocampus and lung samples stored at −80°C were used for HSP-70 protein quantification using western blot. Protein content was measured by BCA assay (Thermo Fisher Scientific, Waltham, MA, USA) according to the standard protocols. The methods used for determination of hippocampus and lung expression of HSP-70 (1:1000; Enzo Life Sciences, Farmingdale, NY, USA) and *β*-actin (1:4000; Santa Cruz Biotechnology, Santa Cruz, CA, USA) were modified from our previous study [12]. Both HSP-70 and actin expressions of both hippocampus and lung were semiquantified using a gel densitometry scanning program. A neutralizing polyclonal rabbit anti-rat HSP-70 polyclonal antibody (25 *μ*g/kg of body weight; ADI-SPA-812-F, Enzo Life Sciences, Inc., Farmingdale, NY, USA) dissolved in nonpyrogenic sterile saline was injected intraperitoneally immediately and once daily for 4 consecutive days after termination of an HAE.

### 2.5. Passive Avoidance Learning Test and Y-Maze Test

We performed a passive avoidance learning trail for each rat at 5 days after each rat had recovered from an HAE, as previously described [13]. A single, inescapable scrambled electric shock was delivered for 3 seconds after the rat entered the dark chamber. The latency to entering the dark compartment from the light compartment was defined as the testing latency. Each rat was scored on Day 5 after HAE.

For determining spatial memory function, the Y-maze test was performed at 5 days after HAE in rats as previously described [14]. For each animal during one Y-maze testing session, both the latency to enter the correct arm (time of reaction) and the number of wrong entries (error number) were measured.

### 2.6. Hippocampal Levels of 2,3-dihydroxybenzoic Acid (2,3-DHBA) and Nitric Oxide Metabolites (NOx)

Hydroxyl radicals (e.g., 2,3-DHBA) were measured using a modified procedure based on the hydroxylation of sodium salicylate by hydroxyl radicals, leading to the formation of 2,3-DHBA [15]. The NOx concentrations in the dialysates obtained from the implanted microdialysis probe in the brain were measured with a NOx analysis system (ENO-20; Eicom, Kyoto, Japan) [16].

### 2.7. Hippocampal Levels of Prooxidant and Antioxidant Enzymes

Hippocampal levels of prooxidant enzymes (e.g., malondialdehyde and oxidized glutathione) and antioxidant enzymes (e.g., reduced glutathione, glutathione peroxidase, glutathione reductase, and superoxide dismutase) were determined. Lipid peroxidation was measured by determining the brain levels of malondialdehyde (MOD) with 2-thiobarbituric acid [17].

The reduced glutathione (GSH; an antioxidant enzyme) and the oxidized glutathione (GSSG; a prooxidant enzyme) were determined as previously described [18, 19]. The glutathione peroxidase (GPx; an antioxidant enzyme), glutathione reductase (GR; an antioxidant enzyme), and superoxide dismutase (SOD; an antioxidant enzyme) in the brain tissues were determined accordingly.

### 2.8. Hippocampal Levels of Proinflammatory Cytokines

The hippocampal levels of inflammatory cytokines (TNF-*α*, IL-10, and IL-1*β*) were quantified using enzyme-linked immunosorbent assay (ELISA) kits (catalogue nos. KRC3011C, BMS629TEN, and ERIL1B, all from Thermo Fisher Scientific Inc., Waltham, MA, USA) specific for rats according to the manufacturers' instructions. The cytokine levels in the brain samples were expressed as pictograms of antigen per milligram of protein.

### 2.9. Measurement of Brain and Lung Water Content

Brain water content (brain edema) or lung water content (pulmonary edema) was evaluated via the wet weight/ dry weight ration method, as previously reported [20–23]. Rats were euthanized and thoroughly exsanguinated before their brains and lungs were excised* en bloc*. Both brain and lung were blot-dried and placed in preweighed trays made of aluminium foil separately. The wet weight of brain tissue or lung tissue was registered immediately on an electronic balance to an accuracy of 0.1 mg. The tray containing brain tissue or lung tissue was then baked in an oven at 100°C for 24 h until a constant weight was achieved. Brain and lung water content were calculated as percentage of [(wet weight-dry weight)/ wet weight] × 100.

### 2.10. Evans Blue Extravasation Assay

Evans blue extravasation assays were conducted in accordance with the methods of Lin et al. [24]. Animals received an intravenous injection of 0.25 ml Evans Blue dye (4%), 3 hours prior to sacrifice. Following that, deeply anesthetized animals were transcardially perfused with ice-cold PBS (100 mL) and both lung and brain were removed, snap-frozen in liquid nitrogen, and stored at −80°C. The quantity of extravasated Evans Blue dye was detected by spectrophotometer (Thermo Fischer Scientific Inc., Waltham, MA, USA) at 610 nm and quantified according to a standard curve. The results are presented as *μ*g of Evans Blue dye per g of tissue.

### 2.11. Measurement of Proinflammatory Cytokines in Bronchoalveolar (BAL) Fluid

The concentrations of interleukin-1*β* (IL-1*β*), IL-6, and tumor necrosis factor-*α* (TNF-*α*) in BAL fluid were determined using ELISA (Thermo Fisher Scientific Inc., Waltham, MA, USA) according to the manufacturer's instruction. Optical densities were read on a plate reader set at 450 nm for IL-6, IL-1*β*, and TNF-*α*. The levels of these cytokines in the samples were calculated from the standard curve multiplied by the dilution factor and were expressed as pictogram per millimetre.

### 2.12. Statistical Analysis

Results are expressed as the mean±SD. Behavioural results are tested for normality (D'Agostino& Pearson omnibus normality test) and skewness using Graph Pal Prism 7.01 (Graph Pad Software, San Diego, CA, USA). The statistical analysis was carried out using repeated measure ANOVA and nonparametric Kruskal-Wallis and Mann-Whitney* U* tests. A statistical *P* value less than 0.05 was considered significant.

## 3. Results

### 3.1. HBO_2_T Attenuated Body Weight Loss and Reduced Water and Food Intake Consumption in HAE Rats

**Figure 2:**
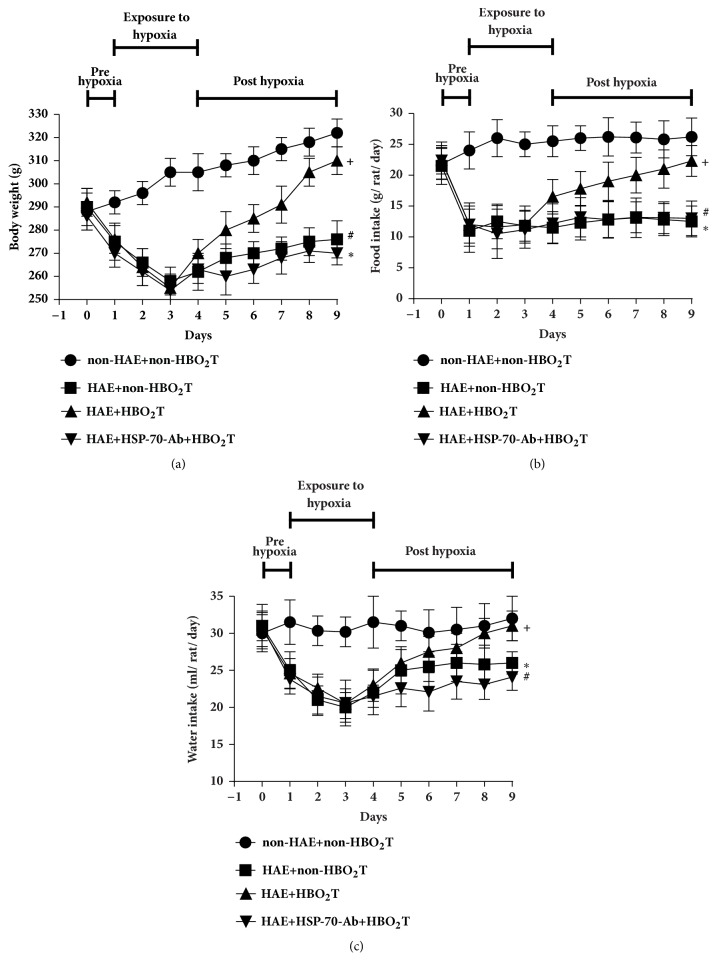
**Mean ± SD body weight in gram (a), food intake in gram (b), and water content in milliliter (c) per day for non-HAE+non-HBO**_2_ **T (●), HAE+non-HBO**_2_ **T (**■**), HAE+HBO**_2_**T (▲), and HAE+HSP-70-Ab+HBO**_2_**T (▼) rats. N=10 per group**. *∗P*<0.05, HAE group versus non-HAE group; +*p*<0.05, HAE+HBO_2_T versus HAE+non-HBO_2_T group; #*p*<0.05, HAE+HSP-70-Ab+HBO_2_T group versus HAE+HBO_2_T group.

During 5 days after HAE, all the values of body weight, food intake, and water intake in the HAE+non-HBO_2_T group were significantly lower than those of the non-HAE+non-HBO_2_T group (Figure 2). However, as compared to those of the HAE+non-HBO_2_T group of rats, the HAE+HBO_2_T group rats had significantly higher values of body weight, food intake, and water intake (Figure 2). The beneficial effects of HBO_2_T were significantly attenuated by HSP-70 Ab treatment (Figure 2).

### 3.2. HBO_2_T Increased Hippocampal and Lung HSP-70 Relative Expression in HAE Rats

**Figure 3:**
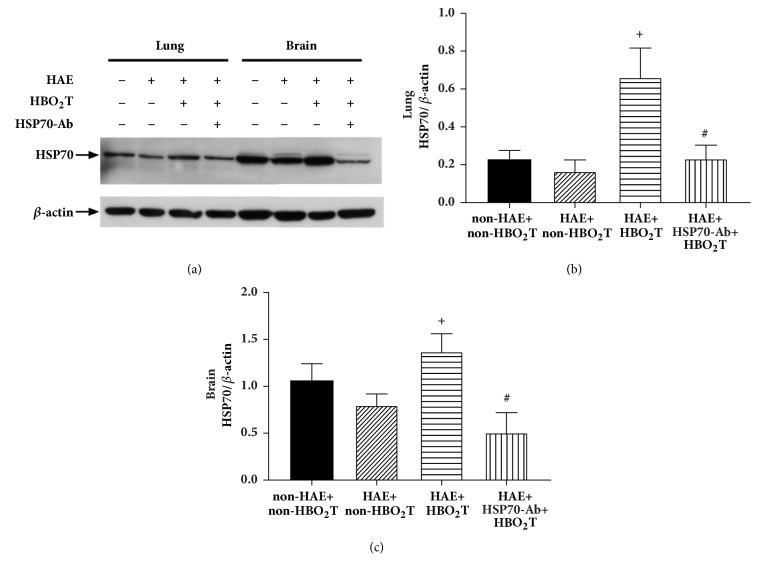
Densitometer analysis of the relative expression of HSP-70 by lung and brain (or hippocampus) tissue samples obtained from the non-HAE rats received non-HBO_2_T (non-HAE+non-HBO_2_T), the HAE rats received non-HBO_2_T (HAE+non-HBO_2_T), the HAE rats received non-HBO_2_T (HAE+non-HBO_2_T), and the HAE rats received HSP-70-Ab and HBO_2_T (HAE+HSP-70-Ab+HBO_2_T). (a) Representative immunoblot of HSP-70. Quantification of (b) lung and (c) brain HSP-70 expression levels compared to *β*-actin control in each groups. *∗p*<0.05 compared with the (HAE+non-HBO_2_T); +*p*<0.05 compared with the HAE+HBO_2_T group. Bars represented the mean ± SD of ten rats per group.

Western blotting analysis revealed that HAE+HBO_2_T group rats had a significantly higher hippocampal and lung expression of HSP-70 than HAE+non-HBO_2_T group rats (p<0.05; Figure 3). In contrast, the HAE+HSP-70 Ab+HBO_2_T group had significantly lower levels of both hippocampal and lung expression of HSP-70 (p<0.05; Figure 3).

### 3.3. HBO_2_T Attenuated Cognitive Deficits in HAE Rats

**Figure 4:**
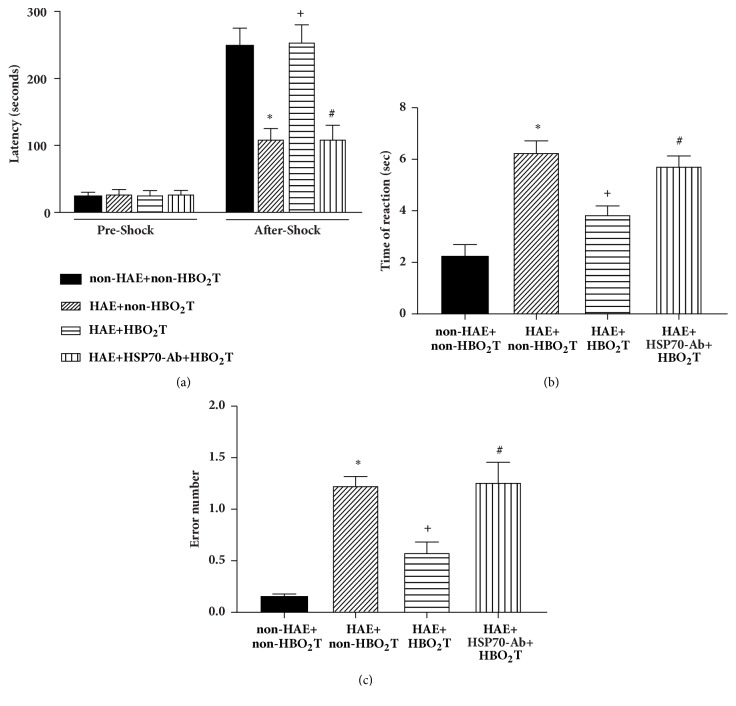
(a) Comparison of latency to entering the dark chamber before receiving the foot shock (preshock; acquisition time) and 24 h after receiving the foot shock (after 24 h; retention time). Each column and bar represents the mean±SD of 10 rats per group. The acquisition time did not significantly differ among the four groups. However, the retention time was significantly lower in the HAE+non-HBO_2_T group compared with the non-HAE+non-HBO_2_T controls at 5 days after injury (*∗P*<0.05). The HAE+HBO_2_T group rats showed a significant increase of retention time in passive avoidance test (^+^*P*<0.05). However, the HAE+HSP-70 Ab+HBO_2_T group rats showed a significant decrease of retention time in passive avoidance compared with the HAE+HBO_2_T group (^#^*P*<0.05). The Y-maze test evaluates learning and spatial memory function in rats 5 days after HAE. Data of both time of reaction (b) and error number (c) are presented as means±SD of n=10 per group. The Y-maze test results showed that the HAE animals had a deficit in spatial memory (time of reaction prolonged and error number increased) compared with the non-HAE+non-HBO_2_T controls at 5 days after injury (*∗P*<0.05). The HAE+HBO_2_T group rats showed a significant decrease of time of reaction and error number in Y-maze test with the HAE+non-HBO_2_T group (^+^*P*<0.05). However, the HAE+HSP-70 Ab+HBO_2_T group rats showed a significant increase of time of reaction and error number in Y-maze test compared with the HAE+HBO_2_T group (^#^*P*<0.05)

The passive avoidance learning tests show both the preshock latency (acquisition time) and 24 h postshock latency (retention time) for all groups in passive avoidance learning test (Figure 4(a)). No between-group differences were found for acquisition time (*P*>0.05) suggesting short-time memory 1.5 hour after test was unaffected by HAE. However, the HAE+non-HBO_2_T group rats had significantly shorter retention time than the non-HAE group of rats (P<0.05). Compared to the HAE+ non-HBO_2_T group of rats, the HAE+HBO_2_T group rats had longer retention time significantly (*P*<0.05). Inhibition of hippocampal HSP-70 expression with HSP-70 Ab significantly attenuated the beneficial effects of HBO_2_T in treating HAE-induced complication (Figure 4(a)).

The Y-maze test shows that HAE+non-HBO_2_T group rats had a deficit in spatial memory (time of reaction prolonged and error number increased) compared with the non-HAE controls at 5 days after injury (Figures 4(b) and 4(c)). Compared to the HAE+non-HBO_2_T group of rats, the HAE+HBO_2_T group rats had better performance in spatial memory (shorter time of reaction and decreased error number) (Figures 4(b) and 4(c)). Again, the beneficial effects of HBO_2_T in treating HAE-induced cognitive deficits were reduced by HSP-70 Ab (Figures 4(b) and 4(c)).

### 3.4. HBO_2_T Reduced Brain and Lung Edema in HAE Rats

**Figure 5:**
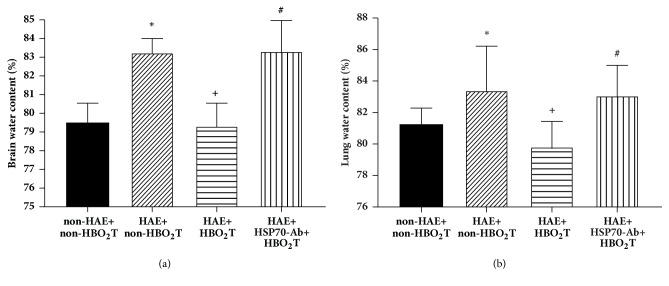
Effect of HBO_2_T on both (a) brain and (b) lung water contents (%) in rats 5 days after HAE. Data are means±SD of n=10 per group. *∗P*<0.05 for HAE+non-HBO_2_T group versus non-HAE+non-HBO_2_T group; ^+^*P*<0.05 for HAE+HBO_2_T group versus HAE+non-HBO_2_T group; ^#^*P*<0.05 for the HAE+HSP-70 Ab+HBO_2_T group versus the HAE+HBO_2_T group.

After 3 days of HAE, both the brain (Figure 5(a)) water content and lung water content (Figure 5(b)) were higher because of edema in the HAE+non-HBO_2_T group rats than in the non-HAE+non-HBO_2_T group rats (*P*<0.05, Figure 5). Brain or lung water content was significantly lower in the HAE+HBO_2_T group rats than in the HAE+non HBO_2_T group rats (*P*<0.05). However, the beneficial effects of HBO_2_T were significantly attenuated in the HAE+HSP-70Ab+HBO_2_T group rats (Figure 5).

**Figure 6:**
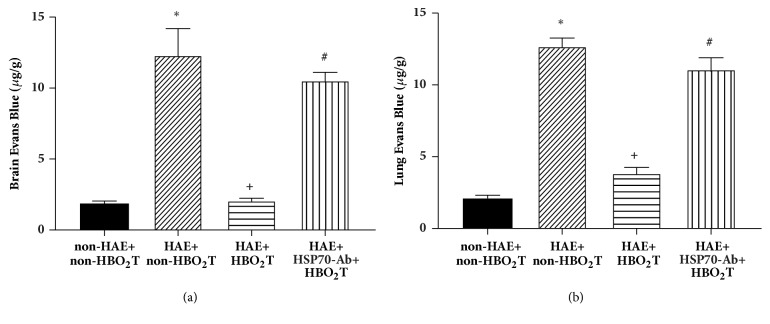
Effects of HBO_2_T on Evans Blue extravasation of both brain (a) and lung (b) tissues in rats 5 days after HAE. Data are represented as the mean ± SD of n=10 per group. *∗P*<0.05 for HAE+non-HBO_2_T group versus non-HAE+ non-HBO_2_T group group; ^+^*P*<0.05 for HAE+HBO_2_T group versus HAE+non-HBO_2_T group; ^#^*P*<0.05 for the HAE+HSP-70 Ab+HBO_2_T group versus HAE+HBO_2_T group.

In order to evaluate the brain-blood-barrier permeability and lung-blood barrier permeability, Evans Blue extravasation assays were conducted after HAE. Significantly, the more extravasated dye was found in both brain and lung specimen of HAE+non-HBO_2_T rats (p<0.05, Figure 6), compared to non-HAE+non-HBO_2_T rats. Treatment with HBO_2_T preserved brain-blood-barrier and lung-blood barrier integrity, which were shown by significantly reduced dye extravasation compared to HAE+non-HBO2T (p<0.05, Figure 6). However, the beneficial effects of HBO_2_T in reducing dye extravasation were significantly reversed by HSP-70 Ab as shown in HAE+HSP-70Ab+HBO_2_T group rats (p<0.05, Figure 6).

### 3.5. HBO_2_T Reversed the Increased Oxidative Stress Markers, Decreased Glutathione System, and Decreased Antioxidant Enzyme in the Brain of HAE Rats

The 2,3-DHBA, NOx, and lipid peroxidation levels in the brain of the HAE+non-HBO_2_T group rats were significantly higher after 3 days of HAE than in the non-HAE+non-HBO_2_T group rats (*P*<0.05, Table 1). Brain levels of these 3 oxidative stress markers were significantly (P<0.05) lower in the HAE+HBO_2_T group rats than in the HAE+non-HBO_2_T group rats (*P*<0.05). The beneficial effects of HBO_2_T were significantly reduced in the HAE+HSP-70- Ab+HBO_2_T group rats (*P*<0.05, Table 1).

### 3.6. HBO_2_T Reversed Both the Decreased GSH System and Decreased Antioxidant Enzyme in the Brain of HAE Rats

Brain expression levels of both GSH (the primary antioxidant in the cell) and GSH/GSSG ratio in the HAE+non-HBO_2_T group rats were significantly (*P*<0.05) lower than in the non-HAE+non-HBO_2_T group rats (Table 1). In contrast, after 3 days of HAE, the hippocampal GSSG (a prooxidant enzyme) was significantly higher (*P*<0.05) in the HAE+non-HBO_2_T group than in the non-HAE+non-HBO_2_T group rats. In HAE rats, HBO_2_T significantly reversed the decreased GSH, the decreased GSH/GSSG, and the increased GSSG levels in the brain (*P*<0.05, Table 1). Again, the beneficial effects of HBO_2_T were significantly attenuated in the HAE+HSP-70 Ab+HBO_2_T group (Table 1). In addition, HBO_2_T significantly reversed post-HAE decreased levels of several antioxidant enzymes including SOD, GPx, and GR in the brain (*P*<0.05; Table 1). The beneficial effects of HBO_2_T were significantly reduced by HSP-70 Ab (*P*<0.05; Table 1).

### 3.7. HBO_2_T Reversed the Increased Brain Levels of Proinflammatory Cytokines in HAE Rats

Levels of cytokines interleukin-1*β* (IL-1*β*), IL-6, interferon- (INF-) *γ*, and tumor necrosis factor- (TNF-) *α* in brain tissue lysate at 5 days after HAE measured using a commercially available ELISA array are summarized in Table 2. All four measured cytokine levels exhibited significant increases in the HAE+non-HBO_2_T group rats than did the non-HAE+non-HBO_2_T control group rats (*P*<0.05; Table 2). HBO_2_T significantly attenuated the HAE-induced brain levels of all four cytokines in the HAE+HBO_2_T group of rats. Again, the beneficial effects of HBO_2_T were significantly reduced by inhibition of brain HSP-70 with HSP-70 Ab in the HAE+HSP-70 Ab+HBO_2_T group rats (Table 2).

### 3.8. HBO_2_T Attenuated Lung Inflammation in HAE Rats

**Figure 7:**
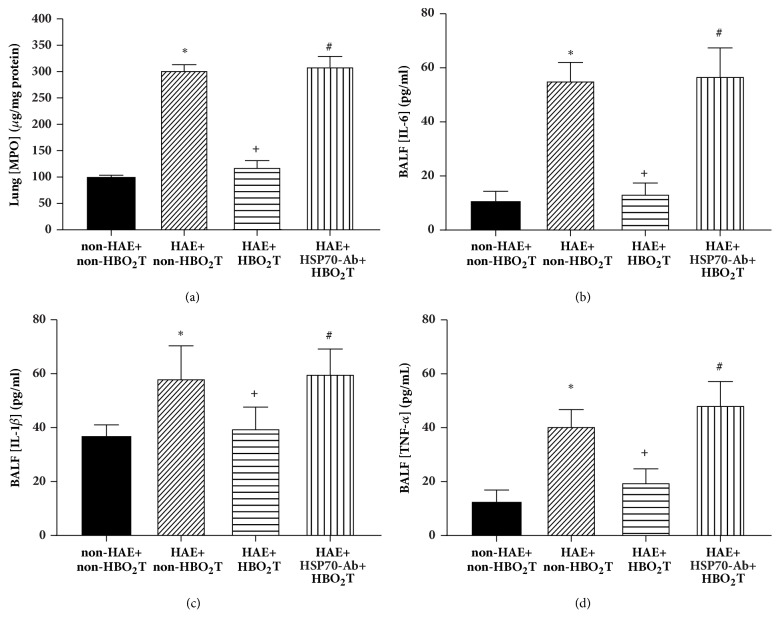
Effect of HBO_2_T on lung levels of (a) myeloperoxidase (MPO) and bronchoalveolar (BAL) fluid levels of (b) interleukin-6 (IL-6), (c) IL-1*β*, and (d) tumor necrosis factor-*α* (TNF-*α*) in rats 5 days after HAE. Data are presented as the means±SD of n=10 per group. *∗P*<0.05 for HAE+ non-HBO_2_T group versus non-HAE+ non-HBO_2_T group; ^+^*P*<0.05 for HAE+HBO_2_T group versus HAE+non-HBO_2_T group; ^#^*P*<0.05 for the HAE+HSP-70 Ab+HBO_2_T group versus HAE+HBO_2_T group.

After 3 days of HAE, the values of BAL fluid levels of MPO (100 *μ*g/ mg protein versus 300 *μ*g/mg protein), IL-6 (8 pg/mL versus 49 pg/mL), IL-1*β* (12 pg/mL versus 65 pg/mL), and TNF-*α* (6 pg/mL versus 49 pg/mL) in the HAE+non-HBO_2_T group were significantly higher than those of the non-HAE+non-HBO_2_T group (*P*<0.05, n=12 each group) (Figure 7). In contrast, as compared with those of the HAE+ non-HBO_2_T group, the HAE+HBO_2_T group had significantly lower values of MPO (120 *μ*g/ mg protein), IL-6 (15 pg/mL), IL-1*β* (19 pg/mL), and TNF-*α* (17 pg/mL) (*P*<0.05, n=12 per group) (Figure 7). The beneficial effects of HBO_2_T were significantly attenuated by HSP-70 Ab treatment (Figure 7).

## 4. Discussion

Our present study tested the protection of HBO_2_T as a therapeutic option for the acute therapy of high-mountain sickness in rats. We found that a regimen of HBO_2_T (100% O_2_ at 2.0 absolute atmospheres for 1 h per day for 5 consecutive days) immediately after HAE injury has protective potential in an animal model of HAE. This study provides the following main findings: (i) the HBO_2_T can increase brain and lung expression of HSP-70; (ii) the HBO_2_T can improve passive avoidance learning and spatial memory dysfunction of the rat after HAE; (iii) the HBO_2_T can attenuate post-HAE cerebral edema; (iv) the HBO_2_T can reverse post-HAE decreased expression of GSH (the main antioxidant in the cell), decreased expression of SOD and GPx (the antioxidant enzymes), and increased GSSG (the main prooxidant in the cell) in the brain; (v) the HBO_2_T can decrease pot-HAE overexpression of proinflammatory cytokines including IL-1*β*, IL-6, IFN-*γ*, and TNF-*α* in the brain; and (vi) the HBO_2_T can attenuate post-HAE induction of pulmonary edema and inflammation. In the present study, we confirm for the first time that post-HAE administration of HBO_2_T attenuates simulated high-mountain sickness syndromes characterized by cerebral and pulmonary edema and the complications. We further elucidate that the beneficial effects of post-HAE HBO_2_T in reducing high-mountain sickness can be blocked by reducing the HSP-70 expression with HSP-70 antibody.

Hyperbaric oxygen therapy is defined as breathing 100% oxygen under increased atmospheric pressure [25]. If descent is impossible, HBO_2_T is highly recommended during the acute phase of the HAE-induced pulmonary edema in patients [26].

Additionally, our present results show that HBO_2_T attenuates HAE-induced brain and pulmonary edema by inducing the HSP-70 expression in rat brain and lung. Our data are consistent with many pervious investigations. For example, a recent study shows that decreasing or increasing HSP-70 exacerbates or attenuates heat-induced cell death, respectively, in rat hypothalamic cells [8]. Stronger HSP-70 immunoreactivity was related to smaller infarction and better functional outcomes [27]. Overexpression of HSP-70 achieved the neuroprotective effect via antiapoptotic mechanisms [28]. HSP-70 protected against apoptotic cell death induced by nuclear factor-*κ*B activation and the nitric oxide synthase II-peroxynitrite signalling cascade in hippocampal CA3 and glial cells [29]. Extracellular HSP-70 can promote neuron death by mediating the production of cytotoxic levels of tumor necrosis factor-alpha [30]. In the present study, HBO_2_T induced an 80% higher expression of HSP-70 in both the brain and lung tissues. Reduction of this relatively mild HSP-70 expression in both the hippocampal and lung tissues with HSP-70 Ab completely reversed the HBO_2_T-induced protection. Our results showed that, after HAE injury, intravenously administrated HSP-70 neutralizing antibodies reduced the expression of antigen in hippocampal and lung tissues. Based on the findings of Liebelt et al. [31], HSP-70 Ab may penetrate into the brain via damaged blood-brain barrier to reduce the beneficial effects of HBO_2_T in brain edema and cognitive deficits by neutralizing HSP-70 produced in the rat hippocampus.

Hyperbaric oxygen therapy has been shown to ameliorate brain injury in a variety of animal models [32]. Hyperbaric oxygen therapy is related to reduced cerebral edema, decreased inflammation, decreased apoptotic cell death, and increased neural regeneration. Indeed, as demonstrated in the present results, HBO_2_T reversed the HAE-induced a decrease of GSH (the main antioxidant in the cell), an increase of GSSG (the main prooxidant in the cell), a decrease of GSH/GSSG, and a decrease in the activity of SOD, GPx, and GR (the antioxidant enzymes) in the brain. In addition, HBO_2_T reversed the HAE-induced upregulation of brain levels of proinflammatory cytokines including IL-1*β*, IL-6, TNF-*α*, and IF-*γ* as well as cognitive deficits. Hypoxia increases brain water content and blood-brain barrier permeability by inducing inflammation [11, 33]. The aquaporin-4 plays a key role in controlling cellular water content as well as cerebral edema formation [34, 35]. These observations suggest that HBO_2_T may inhibit high-altitude cerebral and pulmonary edema by modulating both inflammation and aquaporin-4 levels in situ.

Previous result have shown both food consumption and body weight loss in rats exposed to high altitude compared with rats raised in the low-altitude condition [36, 37]. Highly efficient energy utilization and metabolic homeostasis maintenance rely on hypothalamic neuromodulation [38]. In the present results, HBO_2_T might attenuate HAE-induced reduced food consumption and body weight by attenuating brain edema.

The hypothalamus is a critical regulator of numerous endocrine and autonomic functions [38]. It may be involved in physiological stress responses, blood volume regulation, glucose regulation, thermoregulation, feeding behaviour, and/or circadian rhythms [36]. In the present results, the HAE+non-HBO_2_T rats exhibited brain (including hypothalamus, hippocampus, and others) edema, which led to reduced food consumption, body weight loss, cognitive deficits, and pulmonary edema. Our data suggest that injury of brain tissue is related to the inability to withstand hypobaric hypoxia stress. However, the overexpression of the HSP-70 in the HAE+HBO_2_T rats reduced hypothalamic and/or hippocampus damage from the HAE stress, permitting the HSP-70-mediated HAE+HBO_2_T animals better adaptation.

## 5. Conclusion

In summary, the HAE+non-HBO_2_T rats exhibited brain (including hypothalamus and hippocampus, and others) edema or injury, which might lead to reduced food consumption, body weight loss, cognitive deficits, and pulmonary edema. Brain tissue injury may result in the inability to withstand HAE stress. However, the overexpression of brain HSP-70 in the HAE+HBO_2_T rats reduced brain edema or damage from the HAE stress, permitting the HSP-70-mediated HAE+HBO_2_T animals better adaptation. Our results indicate that HBO_2_T works on a remodeling of brain/ lung to exert a protective effect against simulated high-mountain sickness via enhancing HSP-70 expression in HAE rats.

## Figures and Tables

**Table 1:** Percentage change of oxidative stress markers like 2,3-DHBA, nitric oxide metabolite (NOx), and lipid peroxidation, glutathione system like reduced GSH, GSSG, and ratio of reduced/oxidized glutathione (GSH/GSSG), and antioxidant enzymes like GPx, GR, and SOD levels in the hippocampus of rat brain of all four groups.

Parameters	non-HAE+ non-HBO_2_T	HAE+ non-HBO_2_T	HAE+ HBO_2_T	HAE+HSP-70Ab+ HBO_2_T
2,3-DHBA	100±9	166±14*∗*	118±10^+^	170±16^#^
NOx	101±8	568±17*∗*	212±11^+^	574±21^#^
Lipid peroxidation (MOD)	100±7	297±24*∗*	173±16^+^	303±26^#^
GSH	99±6	75±9*∗*	92±11^+^	72±8^#^
GSSG	101±7	128±11*∗*	90±6^+^	102±9^#^
GSH/GSSG	100±6	65±5*∗*	86±7^+^	64±4^#^
SOD	100±5	70±4*∗*	85±5^+^	68±6^#^
GPx	100±4	79±3*∗*	93±4^+^	77±3^#^
GR	101±3	47±4*∗*	70±5^+^	46±6^#^

Data are mean±SD of 10 rats per group. *∗P*<0.05 for HAE+non-HBO_2_T group versus non-HAE+non-HBO_2_T group; ^+^*P*<0.05 for HAE+HBO_2_T group versus HAE+non-HBO_2_T group; and ^#^*P*<0.05 for HAE+HSP70Ab+HBO_2_T group versus HAE+HBO_2_T group. See Figure 1 legend for group abbreviations.

**Table 2:** Proinflammatory cytokine level expression in the hippocampus of rat brain of all four groups.

Parameters	non-HAE+	HAE+	HAE+	HAE+HSP-70Ab+
(pg/mg protein)	non-HBO_2_T	non-HBO_2_T	HBO_2_T	HBO_2_T
TNF-*α*	31±10	62±12*∗*	39±11^+^	58±13^#^
IL-1*β*	37±11	165±24*∗*	62±12^+^	171±25^#^
IL-6	113±27	448±86*∗*	203±56^+^	459±97^#^
INF-*γ*	24±10	49±21*∗*	22±13^+^	53±24^#^

The cytokine levels are given as mean±SD of 10 rats per group. *∗P*<0.05, compared with non-HBO_2_T group; ^+^*P*<0.05, compared with HAE+ non-HBO_2_T group; and ^#^*P*<0.05, compared with HAE+HBO_2_T group. See Figure 1 legend for group abbreviations.

## Data Availability

The data used to support the findings of this study are available from the corresponding author upon request.

## Abbreviations

HAE: High-altitude exposure
HBO2T: Hyperbaric oxygen therapy
HSP-70: Heat shock protein 70
2,3-DHBA: 2,3-dihydroxybenzoic acid
NOx: Nitric oxide metabolites
MOD: Malondialdehyde
GSH: Reduced glutathione
GSSG: Oxidized glutathione
GPx: Glutathione peroxidase
GR: Glutathione reductase
SOD: Superoxide dismutase
TNF-*α*: Tumor necrosis factor-alpha
IL-1*β*: Interleukin-1 beta
BAL: Bronchoalveolar.
